# Cortisol levels in different tissue samples in posttraumatic stress disorder patients versus controls: a systematic review and meta-analysis protocol

**DOI:** 10.1186/s13643-018-0936-x

**Published:** 2019-01-07

**Authors:** Leigh Luella van den Heuvel, Simonne Wright, Sharain Suliman, Tobias Stalder, Clemens Kirschbaum, Soraya Seedat

**Affiliations:** 1 Department of Psychiatry, Clinical Building, Faculty of Medicine and Health Sciences, University of Stellenbosch, Francie van Zijl Drive, Tygerberg 7505, PO Box 241, Cape Town, 8000 South Africa; 2 Clinical Psychology, University of Siegen, Adolf-Reichwein-Straße 2, 57068 Siegen, Germany; 3 Biological Psychology, TU Dresden, Zellescher Weg 19, 01062 Dresden, Germany

**Keywords:** Posttraumatic stress disorder (PTSD), Trauma, Cortisol

## Abstract

**Background:**

Posttraumatic stress disorder (PTSD) is a disorder that develops following exposure to severely stressful events. Altered cortisol secretion has been reported in PTSD; however, results have been inconsistent. Previous meta-analyses of cortisol levels in PTSD have combined results of studies that have used different tissue samples (blood, saliva, urine) for cortisol measurement and have not included newer methods of determining cortisol levels (e.g. hair samples). In this systematic review, we will synthesise evidence from studies evaluating basal cortisol levels in PTSD patients versus controls and stratify studies according to tissue type used for cortisol measurement. We will also determine whether results from different tissue types can be pooled and if any specific tissue samples have better utility in research studies on PTSD.

**Methods:**

We will perform a systematic review of the scientific literature including all studies that have evaluated basal or baseline cortisol levels in adults with current PTSD versus controls, with and without trauma exposure. Independent reviewers will conduct searches in electronic databases (Medline, CINAHL, PTSDpubs, Web of Science, Scopus, ProQuest Dissertations & Theses A&I, ClinicalTrials.gov, and ICTRP), and additional studies will be obtained by searching the reference lists of articles. Two reviewers (LLvdH and SW) will independently conduct standardised screening, eligibility assessments, data extraction, and quality assessments before qualitative and, if appropriate, quantitative (meta-analysis and meta-regression) synthesis. Disagreements that arise at any stage will be resolved by a third reviewer (ShS).

**Discussion:**

In line with previous reviews, we expect that cortisol levels will be lower in PTSD patients than in controls, but that patterns may vary somewhat according to the tissue sample in which cortisol is measured. This systematic review will assist in developing a better understanding of the acute and chronic patterns of basal cortisol secretion in PTSD and will inform future research.

**Systematic review registration:**

PROSPERO CRD42018091874

**Electronic supplementary material:**

The online version of this article (10.1186/s13643-018-0936-x) contains supplementary material, which is available to authorized users.

## Background

Posttraumatic stress disorder (PTSD) develops following exposure to an extreme stressor(s) or traumatic event(s), such as being confronted with actual or threatened death, serious injury, or sexual violence [1]. Symptoms, causing significant distress or impairment of functioning, persist for at least a month and involve repeated re-experiencing of the traumatic event, avoidance of trauma-related cues, negative changes in thinking and mood, and increased arousal. In the World Health Organization (WHO) World Mental Health (WMH) surveys, the 12-month prevalence of PTSD in the total sample was 1.1% [2] and the estimated lifetime prevalence of PTSD was 2.9% [3]. Of those with PTSD, 42% reported severe role impairment in at least one domain (work, social life, close relationships, or home maintenance) [2] and PTSD was one of the three most disabling disorders [4]. PTSD and other trauma- and stressor-related disorders are distinguished from other psychiatric disorders in their requirement of exposure to stressful events to make a diagnosis [1]. For PTSD, the stressful event(s) must be of a severe or life-threatening nature; PTSD is, therefore, unique in that severe stress plays a central aetiological role in the development of a set of characteristic and persisting symptoms.

The glucocorticoid, cortisol, is generally viewed as the body’s chief stress hormone. Cortisol is synthesised and released from the adrenal cortex which is a component of the neuro-endocrine hypothalamic–pituitary–adrenal (HPA) axis. Cortisol influences processes such as metabolism, immune function, digestion, and behaviour [5]. Cortisol has a baseline diurnal secretion pattern but is also released following exposure to a stressor. It has thus emerged as an objective biological marker of the stress response [5–7]. A negative feedback loop in the HPA axis regulates cortisol secretion and allows for the maintenance of homeostasis. Prolonged or severe stress can lead to dysfunction of the HPA axis with resultant dysregulation of cortisol secretion and associated adverse health outcomes [8, 9]. Cortisol levels can be measured in various tissue samples reflecting cortisol secretion over different time periods. Traditional sampling methods, such as blood (serum and plasma), saliva, and urine are useful for assessing acute cortisol secretion (less than 24 h); however, newer approaches utilising hair and nail samples can provide retrospective and chronic patterns (weeks to months) of cortisol secretion [10–12].

The nature of PTSD as a disorder involving a chronic maladaptive behavioural response to an extreme stressor(s) suggests that it likely has a relationship with a dysregulated endocrine-mediated stress response. Indeed, HPA axis dysregulation has been reported in PTSD; however, results have been inconsistent. A previous systematic review and meta-analysis of basal cortisol levels in PTSD and controls reported no difference in cortisol levels between PTSD patients and controls [13]. The authors pooled results from different tissue samples for their primary analysis and documented significant heterogeneity. In subgroup analysis, they found that plasma and serum cortisol levels were significantly lower in PTSD patients versus trauma unexposed controls (TUC), suggesting that cortisol findings may vary according to tissue type sampled. Furthermore, a recent meta-analysis that only included salivary cortisol levels reported lower cortisol levels in PTSD patients than in controls [14]. Trauma exposure in controls may be another confounding factor, as another meta-analysis that examined the association between trauma exposure in adulthood and cortisol levels found no difference in basal cortisol levels between PTSD patients and trauma-exposed controls (TEC) [15]. Contrary to these findings, a meta-analysis that separately evaluated PTSD patients and PTSD patients with comorbid depression reported lower daily cortisol output for both groups compared to TUC [16]. Inconsistent findings in meta-analyses could also be ascribed to variations in tissue sample type in the included studies and methods used in aggregating results in meta-analysis. Systematic reviews evaluating cortisol levels in PTSD have also not included newer tissue sampling methods, such as hair and nail cortisol measurements. A recent meta-analysis of hair cortisol studies reported lower hair cortisol levels in patients with anxiety disorders (PTSD and generalised anxiety disorder [GAD]) [17], suggesting that adding newer tissue sampling methods could enhance the understanding of HPA axis function in PTSD.

In this systematic review, we aim to bring together all studies that have evaluated basal cortisol levels in PTSD patients versus controls according to the type of tissue sampled. By analysing tissue type, we seek to develop a better understanding of measurement of acute and chronic patterns of basal cortisol secretion in PTSD patients versus controls. We will also seek to establish whether data from different tissue types can be combined and whether sampling a specific tissue for cortisol measurement has greater utility in PTSD studies.

### Objectives

Our objectives are as follows:

1. Our primary objective is to evaluate whether PTSD is associated with altered basal cortisol levels by synthesising the available evidence from primary studies. A previous study that addressed this aim was published more than 10 years ago [13]. Our study involves important differences in terms of methodological approach. In both qualitative and quantitative synthesis (meta-analyses), we will group studies according to the tissue type sampled. See ‘Additional file 1: Table summarising published systematic reviews examining basal cortisol levels in posttraumatic stress disorder’ for a summary of the results of existing systematic reviews and explanation of main differences as compared to this protocol.
2. To evaluate whether pooling results from different tissue samples is meaningful as this may inform future approaches to sampling cortisol.
3. To determine the factors that influence the relationship between basal cortisol levels and PTSD by performing meta-regression where feasible.
4. To perform a critical evaluation of the available literature with a view to identifying areas that require further research.

See Table 1 for research question in PICOTS format.

**Table 1:** Research question in PICOTS format

PICOTS	Inclusion and exclusion criteria
Patients or populations	Adults aged 18 years or older
	Patients with current PTSD based on DSM/ICD criteria
Exposures	Trauma exposure fulfilling DSM/ICD criteria occurring at least a month prior to assessment in PTSD patients and TEC
Comparison group(s)	PTSD patients versus all controls
	Subgroup analysis:
	PTSD patients versus TEC
	PTSD patients versus TUC
Outcomes	Basal or baseline cortisol levels (mean levels and standard deviation) measured in different tissue types
Timing	At least 1 month since trauma exposure in PTSD patients and TEC
Setting	Any setting (inpatient, outpatient, community settings)
Study design	Any study design where cortisol levels are compared in patients versus controls (e.g. cross-sectional, case-control and cohort)

*DSM*, *Diagnostic and Statistical Manual of Mental Disorders*; *ICD*, *International Statistical Classification of Diseases and Related Health Problems*; PICOTS, Population, Intervention, Comparison, Outcomes, Timing, Setting; *PTSD*, posttraumatic stress disorder; *TEC*, trauma-exposed controls; *TUC*, trauma-unexposed controls

## Methods

This protocol was designed in accordance with the guidelines set forth by The Cochrane Collaboration [18] and the Preferred Reporting Items for Systematic Reviews and Meta-Analyses (PRISMA) [19] Statement. The systematic review protocol has been registered with the PROSPERO International prospective register of systematic reviews database (PROSPERO registration number: CRD42018091874) [20]. A PROSPERO search identified two other systematic reviews registered evaluating cortisol levels in PTSD. The first aims to evaluate broader HPA axis function in PTSD, including factors such as dehydroepiandrosterone (DHEA) levels and changes due to psychotherapeutic treatment [21], and the second is focused on evaluating 24-h urinary cortisol levels in PTSD patients and was registered more recently than our systematic review protocol [22]. Two reviewers (LLvdH and SW) will independently conduct standardised screening, eligibility assessments, data extraction, and quality assessments prior to qualitative and quantitative (meta-analyses and meta-regression) synthesis.

### Search strategy

Two independent reviewers (LLvdH and SW) will perform searches in electronic databases (PubMed/MEDLINE, Cumulative Index to Nursing and Allied Health Literature (CINAHL), PTSDpubs, Web of Science, Scopus, and ProQuest Dissertations & Theses A&I) and trial registries (ClinicalTrials.gov and International Clinical Trials Registry Platform [ICTRP]) for published and unpublished studies. Additional studies will be identified by searching the reference lists of relevant reviews and included studies. No limits will be placed on publication date or language; however, articles will only be translated into English and/or authors contacted for information if a study is likely to meet our inclusion criteria conditional upon the title and abstract being available in English. Search terms based on ‘PTSD’ and ‘cortisol’ and applicable synonyms and controlled vocabulary (MeSH terms) will be used where available. The primary search terms will first be formulated in MEDLINE (PubMed) and will then be translated to the other databases. In the databases, all fields will be searched, excluding SCOPUS where title, abstract, and keywords will be searched. We will not place any limitations, such as age group and study design, on searches, but will manually exclude studies according to inclusion and exclusion criteria. We obtained independent peer review from an information specialist who utilised the PRESS methodology [23] and made changes to our search strategy and terms according to their recommendations. The full search terms for each of the databases are included in ‘Additional file 2: Search terms’. Searches in databases will be rerun just prior to analysis to identify any new studies qualifying for inclusion. Searches will be saved and managed utilising the reference manager ‘Mendeley’ where duplicates will be identified and removed. Results from published and unpublished or ‘grey’ literature will be included, provided studies fulfil the inclusion and exclusion criteria and sufficient information is available to assess study quality. Search results will be presented in a PRISMA flowchart according to PRISMA guidelines (Additional file 3: Figure S1 PRISMA flow diagram).

### Selection criteria

#### Study design

Any study design that compares cortisol levels between PTSD patients and controls will be included. The most common designs will be cross-sectional and case–control studies, but cohort and other study designs will also be included, as long as the studies address the main question of this review and fulfil the inclusion/exclusion criteria. If multiple papers related to a single study have been published, the data from the study results will either be combined or the article with the largest sample size will be included.

#### Participants

We will include studies with adults aged 18 years and older that compare cortisol levels between PTSD patients and controls. We will only include patients with current, and not lifetime, PTSD according to DSM/ICD criteria. Controls, with and without, trauma exposure and without a history of prior PTSD will be included. For each tissue type, we will compare cortisol levels in PTSD patients versus all controls (AC). We will also perform subgroup analyses comparing PTSD patients versus TEC and versus TUC. In both patients and TEC, trauma exposure should fulfil DSM/ICD criteria of trauma exposure and should have occurred at least a month prior to assessment. We will include studies where trauma exposure is not clearly defined in our AC group, but not in the subgroup analysis. Including a group of AC, alongside trauma-specified controls, will increase the number of qualifying studies and subgroup analysis will help elucidate the importance of accounting for trauma exposure in controls. We will include studies with medical or psychiatric comorbidity provided the details of comorbidity are well described and comorbidity is not expected to have a substantial impact on outcomes. If possible, psychiatric comorbidity will be included as a factor in meta-regression.

#### Outcomes

To be included in the review, sufficient data to compute effect sizes (mean cortisol levels and standard deviations) must be included in the articles or be made available from the authors on request. Baseline or basal cortisol measurement in any tissue sample type (plasma, serum, whole blood, saliva, urine, hair, nails, and any others) will be included. Different time periods of sampling will be included, such as morning, evening, 24-h output, and different lengths of hair/nail samples. Mean cortisol levels from repeated cortisol measures (e.g. over 24 h) will be included as daily cortisol output. We will exclude studies evaluating cortisol levels in response to psychological or pharmacological stress tests. We will only include studies where the methods to obtain samples (e.g. time of day for acute measures, area on body hair samples obtained from) and to determine cortisol levels (e.g. storage conditions and assay methods used) are clearly described. We will highlight and report separately on studies that utilised more than one tissue sample to determine cortisol levels as these studies may assist in directly evaluating the utility of specific sampling methods in PTSD studies. In our meta-analysis, we will include each study only once and we will prioritise the results according to the largest sample size, the sample with the most complete data, and the sample with the newer tissue sample type (e.g. hair or nails), as the more established sampling approaches have been evaluated in prior meta-analyses.

### Study selection

We will first screen the titles and abstracts of articles and exclude articles based on inclusion/exclusion criteria. We will then screen the full text of the remaining articles and further sort the articles based on inclusion/exclusion criteria. We will utilise an eligibility form to capture and note reasons for including or excluding articles at this stage (Additional file 4: Eligibility form). The eligibility form will be piloted on a subset of studies, and if necessary, modifications will be made. Titles, abstracts, and full texts will be independently reviewed by reviewers 1 and 2 (LLvdH and SW). Any disagreements will be discussed, and if not resolved, eligibility will be determined with the assistance of a third reviewer (ShS). For each stage of review, we will calculate inter-rater agreement utilising the kappa statistic.

Corresponding authors of manuscripts will be contacted via email in the following cases: to obtain a copy of the manuscript if one cannot be obtained; if a manuscript in a foreign language appears eligible for inclusion, the authors will be contacted to enquire whether the details of the study including the results are available in English; if additional information is required to determine study eligibility; to ask for clarification of methods and specific results if these were not included in the manuscript; authors on trial databases will be contacted to enquire whether study details and results are available. If no response is received within 2 weeks, a follow-up email will be sent. If no response is received within another 2 weeks, non-response will be noted and the study excluded. Studies may still be included if authors respond prior to final data analysis.

### Data extraction

Data will be abstracted by two independent reviewers (LLvdH and SW). Data will be entered and managed utilising the Research Electronic Data Capture (REDCap) database application [24]. REDCap is a system for structured, clinical study data capture and is designed to comply with HIPAA regulations. Access control will be password-protected and role-based. The data extraction form will be piloted prior to formal data abstraction, and if necessary, modifications will be made (Additional file 5: Data extraction form). Data extracted will include study characteristics such as study design, setting, and sample size; basic descriptive data (e.g. age, gender, ethnicity) of patients and controls; trauma-related data such as trauma type, trauma load, and duration since trauma; PTSD-related data such as method to determine diagnosis, PTSD severity, duration of illness; physical data such as BMI and blood pressure and any medical or psychiatric comorbidity; cortisol-related data such as tissue type, date and time of sampling, method used to sample and analyse cortisol, mean cortisol levels and standard deviations, and the unit of measurement for patients and controls. If cortisol is measured at multiple time points, we will extract data for each time point and the mean total cortisol over a set period (e.g. 24 h) if available.

#### Quality (risk of bias) assessment

Two reviewers (LLvdH and SW) will perform independent quality or risk of bias (ROB) assessments, and any disagreements will be resolved by a third reviewer (ShS). Inter-rater reliability will be determined (kappa statistic). We will perform ROB assessments with a modified version of the Newcastle–Ottawa scale (NOS) [25] adapted for use in observational studies [26] (Additional file 6: Modified Newcastle Ottawa Scale). The scale assesses for four types of bias (selection bias, performance bias, detection bias, and information bias) with seven questions rated from 0 to 3 with higher scores reflecting lower ROB. In addition, we will pilot a tool for ROB assessment designed for specific use in our review (Additional file 7: Risk of bias (ROB) assessment), based on the guidelines provided by the Agency for Healthcare Research and Quality (AHRQ) [27]. We utilised the approach and applicable components (blinding of outcome, incomplete outcome data, selective reporting) from the Cochrane Risk Of Bias Tool [28] as well as modified components from the NOS. The ROB assessment contains 11 items assessing for selection bias, performance bias, detection bias, attrition bias, reporting bias, and funding or conflict of interest bias. Each item will be rated as ‘low risk of bias’, ‘high risk of bias’, or ‘unclear risk of bias’, and the results for each study will be presented graphically (Additional file 8: Example risk of bias (ROB) figure). The ROB assessment tool will be piloted on a subset of studies, and if required, revisions will be made. We will report on and compare ROB bias assessments obtained with both tools as the modified NOS will allow for comparability with existing studies, whereas the ROB assessment tool designed for our study utilises examples and explanations specific to PTSD and cortisol studies, such as specific methods to determine TE and patient status and includes assessments for additional types of bias not assessed with the modified NOS. We will utilise the Grading of Recommendations, Assessment and Evaluation (GRADE) approach to report the quality of evidence and strength of recommendations [29]. The influence of bias on quantitative outcomes will be assessed by performing sensitivity analysis.

#### Data synthesis

##### Qualitative synthesis

Individual studies will be summarised utilising evidence tables. At a minimum, we will include the authors, year of publication, setting, study design, sample sizes, age, sex, ethnicity, trauma type, time since trauma, trauma and PTSD measures, PTSD severity, PTSD duration, comorbidity, medication use, time or time period of cortisol assessment, cortisol levels, measurement of cortisol levels, and confounders (see ‘Additional file 9: List of potential moderators’ for a full list of moderators that will be included). Data will be organised according to patient and control groups for each study. We will perform a qualitative evaluation of heterogeneity by examining factors such as study design, trauma exposure, settings, populations, and outcome measurements.

##### Quantitative synthesis

Where appropriate, we will perform a meta-analysis for each tissue type sampled. Meta-analysis will only be performed where there are at least two comparable studies with sufficient data available. In articles where results are not reported as means and standard deviations, study authors will be contacted to obtain these summary statistics. If these summary statistics cannot be obtained, we will transform available summary statistics (e.g. median and interquartile range) utilising available formulas [30]. We will compare standardised mean difference (SMD) in cortisol levels between PTSD cases and all controls, and if possible, we will perform a subgroup analysis based on trauma exposure status of controls (TEC and TUC). We will utilise the SMD to allow for pooling of data from studies utilising different methods to determine cortisol (e.g. ELISA versus LC-MS and different tissue samples). Hedge’s *g* will be used for studies with smaller sample sizes. As we expect there to be heterogeneity in study design, we will utilise a random-effects model (DerSimonian and Laird) [31]. Results will be graphically represented utilising forest plots. Heterogeneity will be assessed utilising the Cochrane’s *Q* (chi-squared test) and *I*^2^ statistics and by visually inspecting the forest plots. If significant heterogeneity exists, we will evaluate whether any specific studies significantly influenced the results by excluding each individual study and examining its impact on the pooled SMD and between-study heterogeneity. If there are a sufficient number of studies per tissue type (e.g. ten), we will perform meta-regression, in addition to the subgroup analysis according to trauma exposure status of controls. Potential moderators that will be included in the meta-regression will be year of publication, age, sex, trauma type, time since index trauma, developmental stage of trauma exposure, PTSD severity, psychiatric comorbidity, time period of sampling (e.g. time of day for acute measures and length of hair sample representing retrospective window for hair sampling), and method used to determine cortisol level (ELISA or LC-MS). Moderators will first be entered individually and those with a significance level of 0.1 will be entered in a multivariate meta-regression. The meta-regression will be conducted using a restricted maximum likelihood (REML) model. To address the issue of statistically dependent effect sizes that may arise from multiple methods of cortisol assessment within a study or from either repeated measures within a study that may have a longitudinal design, robust variance estimation procedures in meta-regression will be used. We will evaluate for effect of studies at high ROB and for the effect of study design type by entering the total score and domain sub-scores obtained on the modified NOS and study design type into separate regression models. Further sensitivity analyses will be performed to assess the influence of studies assessed as having a high ROB, by excluding studies with a high ROB and evaluating the impact on main outcomes. We will assess for small study effects with funnel plots (for analyses with 10 or more studies), and statistical tests for asymmetry (Egger test) will be performed where appropriate. If asymmetry is present, we will perform the trim-and-fill procedure [32]. Data will be analysed with STATA IC version 15.

We will present the results for each tissue type sampled in a summary of findings table. As a final step, we will pool the results of studies utilising different tissue types together utilising a cumulative approach to demonstrate trends according to tissue type sampled. We anticipate that we may have to make certain modifications to our meta-analysis approach based on the data collected (e.g. certain important variations may only be evident once the data have been collated). We will stipulate the rationale for any modifications and clearly specify post-hoc analyses.

#### Dissemination of results

The manuscript will be submitted for publication to an appropriate peer-reviewed journal, with preference for an open-access journal to enhance both accessibility and visibility. The results will also be presented at relevant conferences and meetings.

## Discussion

In line with previous reviews, we expect that cortisol levels will generally be lower in PTSD patients than in controls, with larger difference observed when compared to TUC than TEC. We expect that patterns (i.e. the directionality of cortisol levels in PTSD cases compared with controls) will be similar across tissue type but case–control differences may not be statistically significant for all tissue types sampled. Some of the variability may be the result of the time window of cortisol measurement, as reflected by the tissue type sampled. Systematically identifying this variability may assist in better delineating acute and chronic patterns of basal cortisol secretion in PTSD. We will utilise outcomes of this review to identify aspects requiring additional investigation as well as provide suggestions as to which tissue sample types may have better utility for PTSD studies.

## Additional files

Additional file 1: Table summarising published systematic reviews examining basal cortisol levels in posttraumatic stress disorder. (DOCX 17 kb)

Additional file 2: Search terms. (DOCX 14 kb)

Additional file 3: **Figure S1.** PRISMA flow diagram. (DOC 29 kb)

Additional file 4: Eligibility form. (PDF 47 kb)

Additional file 5: Data extraction form. (PDF 92 kb)

Additional file 6: Modified Newcastle Ottawa Scale. (PDF 40 kb)

Additional file 7: Risk of bias (ROB) assessment. (PDF 50 kb)

Additional file 8: Example risk of bias (ROB) figure. (DOCX 13 kb)

Additional file 9: List of potential moderators. (DOCX 15 kb)

Additional file 10: PRISMA-P Checklist. (DOCX 30 kb)

## Abbreviations

AC: All controls
AHRQ: Agency for Healthcare Research and Quality
BMI: Body mass index
DSM: *Diagnostic and Statistical Manual of Mental Disorders*
ELISA: Enzyme-linked immunosorbent assay
GAD: Generalised anxiety disorder
HIPAA: Health Insurance Portability and Accountability Act of 1996
HPA: Hypothalamic–pituitary–adrenal
ICD: *International Statistical Classification of Diseases and Related Health Problems*
LC-MS: Liquid chromatography–mass spectrometry
NOS: Newcastle–Ottawa scale
PRISMA: Preferred Reporting Items for Systematic Reviews and Meta-Analyses
PTSD: Posttraumatic stress disorder
REDCap: Research Electronic Data Capture
ROB: Risk of bias
TEC: Trauma-exposed controls
TUC: Trauma-unexposed controls
WHO: World Health Organization
WMH: World Mental Health
