# Supplementary material for: Cortisol levels in different tissue samples in posttraumatic stress disorder patients versus controls: a systematic review and meta-analysis protocol
Source: Syst Rev. 2019 Jan 7;8:7. doi: 10.1186/s13643-018-0936-x (PMC6322257; doi:10.1186/s13643-018-0936-x)
Supplement: Supplementary file 1 — Table summarising published systematic reviews examining basal cortisol levels in posttraumatic stress disorder. (DOCX 17 kb) [file 13643_2018_936_MOESM1_ESM.docx]

**Additional file 1: Table summarising published systematic reviews examining basal cortisol levels in posttraumatic stress disorder**

| **Study** | **Main aims** | **Type of study** | **Populations** | **Tissue type (s) included** | **Included papers and main results** |
| --- | --- | --- | --- | --- | --- |
| Meewise et al, 2007 [13]^a^ | To compare basal cortisol levels in adults with current PTSD and in people without psychiatric disorder | SR & MA | PTSD versus AC  Subgroup analysis:   - PTSD versus TUC - PTSD versus TEC | Plasma, saliva, serum & urine | 37 papers included (828 participants with PTSD and 800 controls)  Main results:   - No difference between PTSD patients and controls - No difference based on tissue type (plasma/serum or saliva or urine)   Subgroup analysis (only in plasma/serum samples):   - Cortisol levels lower in PTSD patients than in TUC - Cortisol levels lower in PTSD patients with PTSD secondary to sexual or physical abuse, in female samples and if PM sampling used - No difference based on years since trauma, depression comorbidity, year of publication |
| Klaassens et al, 2012 [15]^b^ | To determine whether trauma exposure during adulthood is association with HPA-axis dysregulation in the absence or presence of PTSD | MA | TEC versus TUC  TEC versus PTSD (TE – specifically TE in adulthood) | Plasma, saliva, & urine (included DT) | 37 papers included (1120 TEC, 508 TUC, and 840 PTSD patients)  Main results:   - No difference in cortisol levels in TEC versus PTSD patients (34 studies) or versus TUC   Subgroup analysis:   - No difference between TEC and PTSD patients according to tissue type, type of trauma, gender, age group, lifetime psychiatric disorders in control subjects, medication use or comorbid MDD in PTSD patients, or study quality. |
| Morris et al, 2012 [16]^c^ | To examine changes in the HPA-axis between PTSD patients (with and without MDD comorbidity), TEC and TUC | SR, MA & MR | PTSD versus TUC  PTSD+MDD versus TUC  TEC versus TUC | CSF, plasma, saliva, serum & urine | 47 studies included (2521 TEC, 2746 TUC, 504 PTSD patients & 237 PTSD+MDD patients)  Main results   - AM levels were lower in PTSD and PTSD+MDD than TUC - PM levels were lower in PTSD and TEC than TUC and higher in PTSD+MDD than TUC - Daily output levels were lower in PTSD and PTSD+MDD than in TUC   Aggregate effects sizes   - AM levels were lower in PTSD+MDD than in PTSD and TEC and lower in PTSD than in TEC - PM levels were higher in PTSD+MDD than in PTSD and TEC   Meta-regression and meta-ANOVA   - AM levels were lower for saliva versus plasma in TEC and higher in saliva versus plasma for PTSD+MDD and were positively associated with childhood trauma exposure in PTSD+MDD - PM levels were lower for plasma versus saliva in PTSD and TEC, were negatively associated with more males in TEC, and negatively associated with time since trauma in PTSD - Daily output levels were positively associated with age in PTSD+MDD, negatively associated with increased males in PTSD, and negatively associated with time since trauma in PTSD and PTSD+MDD |
| Pan et al, 2018 [14]^d^ | To compare salivary cortisol concentration levels in  PTSD patients versus controls | SR, MA & MR (used to assess for heterogeneity) | PTSD versus AC  PTSD versus TUC | Saliva | 22 papers included (1064 participants with PTSD and 2322 controls)  Main results:   - Lower salivary cortisol levels in PTSD patients than in AC and TUC   Subgroup analyses:   - Lower salivary cortisol levels in PTSD patients than controls if studies were completed after 2007 (versus before 2007) and if samples were collected in the AM (versus PM)   Meta-regression:   - Included 8 variables (country, PTSD assessment, collection time, publication year, whether study reported on assay methods, inter-assay variation, intra-assay variation, sensitivity, freeze temperature) - Whether PTSD was assessed with the CAPS or another measure was the only source of heterogeneity on the MR |
| ^a^ Systematic review most similar to our protocol, but was published more than 10 years ago and newer tissue sampling measures not included. Study pooled results from different tissue samples for main outcomes and subgroup analysis was only done on plasma and serum samples, whereas our study will be performing meta-analysis according to tissue sample type separately.  ^b^ Main focus of the meta-analysis was to examine whether adulthood trauma exposure was associated with altered HPA-axis function. Only studies with adulthood trauma exposure were included and primary group of interest was TEC and thus for instance studies evaluating PTSD versus TUC were not included.  ^c^ Systematic review separated groups according to MDD comorbidity and thus PTSD groups will be differently organised than our study. All groups were also compared to TUC and thus studies directly comparing PTSD versus TEC were not included in the meta-analysis and aggregate effect sizes compared to TUC were used instead.  ^d^ Systematic review focused on one tissue sample type, namely salivary cortisol levels and was published after this systematic review protocol was formulated and registered.  AC, all controls; AM, morning samples; CAPS, clinician administered PTSD scale; CSF, cerebrospinal fluid; DT, dynamic tests of HPA-axis reactivity; HPA, hypothalamic-pituitary-adrenal; MA, meta-analysis; MDD, major depressive disorder; MR, meta-regression; PM, afternoon samples; PTSD, posttraumatic stress disorder; SR, systematic review; TEC, trauma exposed controls; TUC, trauma unexposed controls | | | | | |
