# Supplementary material for: Cortisol levels in different tissue samples in posttraumatic stress disorder patients versus controls: a systematic review and meta-analysis protocol
Source: Syst Rev. 2019 Jan 7;8:7. doi: 10.1186/s13643-018-0936-x (PMC6322257; doi:10.1186/s13643-018-0936-x)
Supplement: Supplementary file 2 — Search terms. (DOCX 14 kb) [file 13643_2018_936_MOESM2_ESM.docx]

**Additional File 2: Search terms**

| **Database** | **Full search term** |
| --- | --- |
| Pubmed Medline | ((("Stress Disorders, Post-Traumatic"[Mesh]) OR ((post traum* stress OR post-traum* stress OR posttraum* stress OR PTSD)))) AND (("Adrenal Cortex Hormones"[Mesh]) OR ((((((adrenal* hormone*) OR *corticoster*) OR *corticoi*) OR glucocort*) OR *hydrocort*) OR *cortisol)) |
| CINAHL | ( (MH "Stress Disorders, Post-Traumatic+") OR TX ( "post traum* stress" OR "post-traum* stress" OR "posttraum* stress" OR PTSD ) ) AND ((MH "Adrenal Cortex Hormones+") OR ("adrenal* hormone*" OR *corticoster* OR *corticoi* OR glucocort* OR *hydrocort* OR *cortisol)) |
| PTSDpubs | MAINSUBJECT.EXACT.EXPLODE("PTSD") OR ((post traum* stress) OR (post-traum* stress) OR (posttraum* stress) OR PTSD)) AND (MAINSUBJECT.EXACT.EXPLODE("Glucocorticoids") OR ((adrenal* hormone* ) OR corticoster* OR corticoi* OR glucocort* OR hydrocort* OR cortisol)) |
| Web of Science | (("post traum* stress" OR "post-traum* stress" OR "posttraum* stress" OR PTSD) AND ("adrenal* hormone*" OR *corticoster* OR *corticoi* OR glucocort* OR *hydrocort* OR *cortisol)) |
| Scopus | ( TITLE-ABS-KEY ( "post traum* stress" OR "post-traum* stress" OR "posttraum* stress" OR ptsd ) ) AND ( TITLE-ABS-KEY ( "adrenal* hormone*" OR *corticoster* OR *corticoi* OR glucocort* OR *hydrocort* OR *cortisol ) ) |
| ProQuest Dissertations & Theses A&I‎ | (MAINSUBJECT.EXACT("Post traumatic stress disorder") OR “post traum* stress” OR “post-traum* stress” OR “posttraum* stress” OR PTSD) AND (MAINSUBJECT.EXACT("Steroids") OR “adrenal* hormone*” OR corticoster* OR corticoi* OR glucocort* OR hydrocort* OR cortisol) |
| ClinicalTrials.gov | (“post traumatic stress” OR “posttraumatic stress” OR “post-traumatic stress” OR PTSD) AND (“adrenal hormone” OR corticosteroid OR corticoid OR glucocorticoid OR hydrocortisone OR cortisol) |
| ICTRP | hormone OR post traumatic stress AND corticoster* OR post traumatic stress AND corticoi* OR post traumatic stress AND cortisol OR post traumatic stress AND glucocort* OR post traumatic stress AND hydrocort* OR posttraumatic stress AND adrenal hormone OR posttraumatic stress AND corticoster* OR posttraumatic stress AND corticoi* OR posttraumatic stress AND cortisol OR posttraumatic stress AND glucocort* OR posttraumatic stress AND hydrocort* OR post-traumatic stress AND adrenal hormone OR post-traumatic stress AND corticoster* OR post-traumatic stress AND corticoi* OR post-traumatic stress AND cortisol OR post-traumatic stress AND glucocort* OR post-traumatic stress AND hydrocort* OR PTSD AND adrenal hormone OR PTSD AND corticoster* OR PTSD AND corticoi* OR PTSD AND cortisol OR PTSD AND glucocort* OR PTSD AND hydrocort* |
