# Supplementary material for: Cortisol levels in different tissue samples in posttraumatic stress disorder patients versus controls: a systematic review and meta-analysis protocol
Source: Syst Rev. 2019 Jan 7;8:7. doi: 10.1186/s13643-018-0936-x (PMC6322257; doi:10.1186/s13643-018-0936-x)
Supplement: Supplementary file 3 — Figure S1. PRISMA flow diagram. (DOC 29 kb) [file 13643_2018_936_MOESM3_ESM.doc]

**Additional file 3: Figure 1 PRISMA flow diagram**

**Screening**

**Included**

**Eligibility**

**Identification**

Records identified through database searching
(n = )

Additional records identified through other sources
(n = )

Records after duplicates removed
(n = )

Records screened
(n = )

Records excluded
(n = )

Full-text articles assessed for eligibility
(n = )

Full-text articles excluded, with reasons
(n = )

Studies included in qualitative synthesis
(Total; n = )

(Plasma; n = )

(Serum; n = )…

Studies included in quantitative synthesis (meta-analysis)
(Total; n = )

(plasma; n = )

(Serum; n = )…
