# Supplementary material for: Cortisol levels in different tissue samples in posttraumatic stress disorder patients versus controls: a systematic review and meta-analysis protocol
Source: Syst Rev. 2019 Jan 7;8:7. doi: 10.1186/s13643-018-0936-x (PMC6322257; doi:10.1186/s13643-018-0936-x)
Supplement: Supplementary file 4 — Eligibility form. (PDF 47 kb) [file 13643_2018_936_MOESM4_ESM.pdf]

Eligibility form

Record ID ______________________________

Reviewer initials: ______________________________

Date form completed ______________________________
(dd/mm/yyyy)

Study design

1. Any study design comparing cortisol levels between PTSD patients and controls? (only original studies, not reviews)
◯ Yes
◯ No
◯ Unclear

Notes (can include quotes from the text, location in the text and reviewer explanations) ______________________________

Participants

2. Study in adults, aged 18 years and older?
◯ Yes
◯ No
◯ Unclear

Notes (can include quotes from the text, location in the text and reviewer explanations) ______________________________

3. PTSD patients with current PTSD according to DSM/ICD criteria?
◯ Yes
◯ No
◯ Unclear

Notes (can include quotes from the text, location in the text and reviewer explanations) ______________________________

Exposures

4. Trauma exposure fulfilling DSM/ICD criteria occurred at least a month prior to assessment? (in patients and trauma exposed controls)
◯ Yes
◯ No
◯ Unclear

Notes (can include quotes from the text, location in the text and reviewer explanations) ______________________________

5. Controls without a history of prior PTSD (lifetime PTSD)?
◯ Yes
◯ No
◯ Unclear

Notes (can include quotes from the text, location in the text and reviewer explanations)

____

Comparisons

6. Cortisol levels compared between which groups (tick all that apply)

- ☐ PTSD patients and trauma exposed controls (TEC)
- ☐ PTSD patients and trauma unexposed controls (TUC)
- ☐ PTSD patients and all controls (both trauma exposed and trauma unexposed)
- ☐ Other
- ☐ Unclear/not stated

Notes (can include quotes from the text, location in the text and reviewer explanations)

____

Outcomes

7. Study assesses baseline or basal cortisol levels? (Not psychological or pharmacological stress tests)

- ◯ Yes
- ◯ No
- ◯ Unclear

Notes (can include quotes from the text, location in the text and reviewer explanations)

____

8. Sufficient data to compute effects sizes (mean cortisol levels and standard deviations in patients and controls) stated in articles or could possibly be obtained from authors?

- ◯ Yes
- ◯ No
- ◯ Unclear

Notes (can include quotes from the text, location in the text and reviewer explanations)

____

Timing

9. All relevant measures completed at least a month since trauma exposure (PTSD diagnostic status, cortisol levels)?

- ◯ Yes
- ◯ No
- ◯ Unclear

Notes (can include quotes from the text, location in the text and reviewer explanations)

____

Reviewer decision

Include study in the review?

- ◯ Yes
- ◯ No
- ◯ Unclear/uncertain

Notes (can include quotes from the text, location in the text and reviewer explanations)

____

Is additional information required from the study authors before a final assessment can be made?	◯ Yes<br>◯ No
What information is required before a decision can be made	__________________________________

To be completed after both reviewers have completed individual elligibility review

Do both reviewers agree?	◯ Yes<br>◯ No<br>◯ Unclear/uncertain
Notes (can include quotes from the text, location in the text and reviewer explanations)	__________________________________
Third reviewer decision (if both reviewers don't agree)	◯ Include<br>◯ Exclude
Study included?	◯ Yes<br>◯ No
Reason for exclusion	__________________________________
Retain study for:	☐ Background/discussion<br>☐ Review of references<br>☐ Other
Notes	__________________________________
