# Supplementary material for: Cortisol levels in different tissue samples in posttraumatic stress disorder patients versus controls: a systematic review and meta-analysis protocol
Source: Syst Rev. 2019 Jan 7;8:7. doi: 10.1186/s13643-018-0936-x (PMC6322257; doi:10.1186/s13643-018-0936-x)
Supplement: Supplementary file 5 — Data extraction form. (PDF 92 kb) [file 13643_2018_936_MOESM5_ESM.pdf]

Study Information

Record ID

____

Study ID:

____
((surname of first author and year first full report of study was published e.g. Smith 2001))

Report ID
(if there are multiple reports of this study)

____
((surname of first author and year this report was published e.g. Smith 2001))

Report IDs of other reports of this study (e.g. duplicate publications, follow-up studies)

____
(Multiple reports of the same study need to be linked together)

Reviewer initials:

____

Date form completed

____
(dd/mm/yyyy)

Manuscript/report Title: (title of paper/ abstract/ report that data are extracted from)

____

Authors:

____

Journal:

____

Publication date

____

Page numbers:

____

DOI:

____

Reference details/citation (any other reference details not entered above)

____

Report author contact details (add all details listed for the authors)

____

Publication type	◯ Full report<br>◯ Brief report<br>◯ Letter<br>◯ Abstract<br>◯ Conference report<br>◯ Book chapter<br>◯ Other<br>◯ Not stated/unclear
Details regarding publication type	__________________________________
Study funding source (including role of funders)	____
Possible conflicts of interest (for study authors)	__________________________________
Notes:	____

Eligibility form

Reviewer initials: ______________________

Date form completed ______________________ (dd/mm/yyyy)

Study design

1. Any study design comparing cortisol levels between PTSD patients and controls? (only original studies, not reviews)	◯ Yes<br>◯ No<br>◯ Unclear
Notes (can include quotes from the text, location in the text and reviewer explanations)	______________________

Participants

2. Study in adults, aged 18 years and older?	◯ Yes<br>◯ No<br>◯ Unclear
Notes (can include quotes from the text, location in the text and reviewer explanations)	______________________
3. PTSD patients with current PTSD according to DSM/ICD criteria?	◯ Yes<br>◯ No<br>◯ Unclear
Notes (can include quotes from the text, location in the text and reviewer explanations)	______________________

Exposures

4. Trauma exposure fulfilling DSM/ICD criteria occurred at least a month prior to assessment? (in patients and trauma exposed controls)	◯ Yes<br>◯ No<br>◯ Unclear
Notes (can include quotes from the text, location in the text and reviewer explanations)	______________________
5. Controls without a history of prior PTSD (lifetime PTSD)?	◯ Yes<br>◯ No<br>◯ Unclear
Notes (can include quotes from the text, location in the text and reviewer explanations)	______________________

Comparisons

6. Cortisol levels compared between which groups (tick all that apply)	☐ PTSD patients and trauma exposed controls (TEC)<br>☐ PTSD patients and trauma unexposed controls (TUC)<br>☐ PTSD patients and all controls (both trauma exposed and trauma unexposed)<br>☐ Other<br>☐ Unclear/not stated
Notes (can include quotes from the text, location in the text and reviewer explanations)	__________________________________

Outcomes

7. Study assesses baseline or basal cortisol levels? (Not psychological or pharmacological stress tests)	◯ Yes<br>◯ No<br>◯ Unclear
Notes (can include quotes from the text, location in the text and reviewer explanations)	__________________________________
8. Sufficient data to compute effects sizes (mean cortisol levels and standard deviations in patients and controls) stated in articles or could possibly be obtained from authors?	◯ Yes<br>◯ No<br>◯ Unclear
Notes (can include quotes from the text, location in the text and reviewer explanations)	__________________________________

Timing

9. All relevant measures completed at least a month since trauma exposure (PTSD diagnostic status, cortisol levels)?	◯ Yes<br>◯ No<br>◯ Unclear
Notes (can include quotes from the text, location in the text and reviewer explanations)	__________________________________

Reviewer decision

Include study in the review?	◯ Yes<br>◯ No<br>◯ Unclear/uncertain
Notes (can include quotes from the text, location in the text and reviewer explanations)	__________________________________
Is additional information required from the study authors before a final assessment can be made?	◯ Yes<br>◯ No

What information is required before a decision can be made

____

To be completed after both reviewers have completed individual elligibility review

Do both reviewers agree?

- ◯ Yes
- ◯ No
- ◯ Unclear/uncertain

Notes (can include quotes from the text, location in the text and reviewer explanations)

____

Third reviewer decision (if both reviewers don't agree)

- ◯ Include
- ◯ Exclude

Study included?

- ◯ Yes
- ◯ No

Reason for exclusion

____

Retain study for:

- ☐ Background/discussion
- ☐ Review of references
- ☐ Other

Notes

____

Population And Setting

Population and setting
Include comparative information for each group (i.e. controls) if available.
Can include quotes from the text.

1. Country and City of study

____

Notes for above (can include quotes from the text, location in the text and reviewer explanations)

____

2. Population description
(from which study participants are drawn)

____

Notes for above (can include quotes from the text, location in the text and reviewer explanations)

____

3. Setting
(including location and social context)

____

Notes for above (can include quotes from the text, location in the text and reviewer explanations)

____

4. Socio-economic status of participants

____

Notes for above (can include quotes from the text, location in the text and reviewer explanations)

____

5. Inclusion cirteria:

____

Notes for above (can include quotes from the text, location in the text and reviewer explanations)

____

6. Exclusion criteria:

____

Notes for above (can include quotes from the text, location in the text and reviewer explanations)

____

7. Method/s of recruitment of participants

____

Notes for above (can include quotes from the text, location in the text and reviewer explanations)

____

8. Informed consent obtained

- ◯ Yes
- ◯ No
- ◯ Unclear/not stated

Notes for above (can include quotes from the text, location in the text and reviewer explanations)

____

Notes

____

Study Methods

Study methods

Descriptions as stated in report/paper. Can include quotes from the text.

1. Aim(s) of study

____

Notes for above (can include quotes from the text, location in the text and reviewer explanations)

____

2. Study design

◯ Cross-sectional study
◯ Case-control study
◯ Cohort study
◯ Clinical trial
◯ Other design type
◯ Not reported/unclear

Notes for above (can include quotes from the text, location in the text and reviewer explanations)

____

3. Start date:

____

4. End date:

____

5. Duration of participation (from recruitment to last follow-up)

____

Notes for above (can include quotes from the text, location in the text and reviewer explanations)

____

6. Method used to determine trauma exposure (in patients and controls)

____

Notes for above (can include quotes from the text, location in the text and reviewer explanations)

____

7. Method used to determine PTSD diagnostic status (in patients and controls)

____

Notes for above (can include quotes from the text, location in the text and reviewer explanations)

____

8. Method used to assess for comorbidity (in patients and controls)	__________________________________
Notes for above (can include quotes from the text, location in the text and reviewer explanations)	__________________________________
10. Number of study groups/arms	____________________________
11. Types of control groups included (Tick all that apply)	☐ Trauma exposed controls<br>☐ Trauma unexposed controls<br>☐ Combined trauma exposed and unexposed controls<br>☐ Trauma exposure of controls not determined/defined<br>☐ Other types of controls
Notes for above (can include quotes from the text, location in the text and reviewer explanations)	__________________________________
12. Ethical approval obtained for study	◯ Yes<br>◯ No<br>◯ Unclear/Not stated
Notes for above (can include quotes from the text, location in the text and reviewer explanations)	__________________________________
Notes	__________________________________

Modified Newcastle-Ottawa Scale (NOS)

Domain of evaluation: Methods for selecting study participants (i.e. Selection bias)

Is the source population (cases, controls, cohorts) appropriate and representative of the population of interest?<br>Example of low risk of bias: A consecutive sample or random selection from a population that is representative of the condition under study.<br>Example of moderate risk of bias: A consecutive sample or random selection from a population that is not highly representative of the condition under study.<br>Example of high risk of bias: The source population cannot be defined or enumerated (i.e. volunteering or self-recruitment).	◯ Definitely no (high risk of bias)<br>◯ Mostly no<br>◯ Mostly yes<br>◯ Definitely yes (low risk of bias)

Domain of evaluation: Methods to control confounding (i.e. Performance bias)

Is the sample size adequate and is there sufficient power to detect a meaningful difference in the outcome of interest?<br>Example of low risk of bias: Sample size was adequate and there was sufficient power to detect a difference in the outcome.<br>Example of high risk of bias: Sample size was small and there was not enough power to test outcome of interest.	◯ Definitely no (high risk of bias)<br>◯ Mostly no<br>◯ Mostly yes<br>◯ Definitely yes (low risk of bias)
Did the study identify and adjust for any variables or confounders that may influence the outcome?<br>Example of low risk of bias: The study identified and adjusted for all possible confounders that may influence estimates of association between exposure and outcome (i.e. Was the patient being treated for a medical condition such as chronic pain and was being prescribed opioids while on methadone treatment?)<br>Example of moderate risk of bias: The study identified and reported possible variables that may influence the outcome but did not explore the interaction.<br>Example of high risk of bias: The study either did not report any variables of influence or acknowledge variables of influence when it was clear they were present.	◯ Definitely no (high risk of bias)<br>◯ Mostly no<br>◯ Mostly yes<br>◯ Definitely yes (low risk of bias)

Domain of evaluation: Statistical methods (i.e. Detection bias)

Did the study use appropriate statistical analysis methods relative to the outcome of interest?
Example of low risk of bias: The study reported use of appropriate statistical analysis as required (i.e. adjusting for an unbalanced distribution of a specific covariate among sexes, or correcting for multiple testing error)
Example of moderate risk of bias: The study either used correct statistical methods but did not report them well, or used the incorrect methods but reported them in detail.
Example of high risk of bias: The study did not use appropriate statistical analysis as required (i.e. did not adjust for an unbalanced distribution of a specific covariate among sexes, or correct for multiple testing error when necessary) or did not report them adequately.

- ◯ Definitely no (high risk of bias)
- ◯ Mostly no
- ◯ Mostly yes
- ◯ Definitely yes (low risk of bias)

Is there little missing data and did the study handle it accordingly?
Example of low risk of bias: The study acknowledged missing data to be less than 10% and specified the method of handling it.
Example of moderate risk of bias: The study either had greater than 15% but they specified the method they used to handle it.
Example of high risk of bias: The study had greater than 15% missing data and did not handle it at all.

- ◯ Definitely no (high risk of bias)
- ◯ Mostly no
- ◯ Mostly yes
- ◯ Definitely yes (low risk of bias)

Domain of evaluation: Methods for measuring outcome variables (i.e. Information bias)

Is the methodology of the outcome measurement explicitly stated and is it appropriate?
Example of low risk of bias: The study provides a detailed description of the outcome measure(s) which are appropriate for the outcome of interest.
Example of moderate risk of bias: The study provides a somewhat complete description of outcome measurements and they are justified.
Example of high risk of bias: The study provides limited information on the methods of measuring the outcome and the measure is not appropriate considering the outcome.

- ◯ Definitely no (high risk of bias)
- ◯ Mostly no
- ◯ Mostly yes
- ◯ Definitely yes (low risk of bias)

Is there an objective assessment of the outcome of interest?
Example of low risk of bias: The study used objective methods to discern the outcome status of participants (i.e. laboratory measurements, medical records).
Example of moderate risk of bias: The study relied on subjective data as the primary method to discern outcome status of participants (i.e. self-report).
Example of high risk of bias: The study had limited reporting about assessment of outcomes.

- ◯ Definitely no (high risk of bias)
- ◯ Mostly no
- ◯ Mostly yes
- ◯ Definitely yes (low risk of bias)

Risk of bias (ROB) assessment

Reviewer initials: ________________________________

Date form completed ________________________________
(dd/mm/yyyy)

Selection bias
Selection bias refers to systematic differences between baseline characteristics of the groups that are compared

1. Ascertainment of trauma exposure
Was trauma exposure based on DSM/ICD criteria and assessed using a valid and reliable measure and was the same method of ascertainment utilised in cases and controls?

- ◯ Low risk of bias - Trauma exposure was based on DSM/ICD criteria AND assessed with a validated trauma assessment measure (e.g. Life Events Checklist for DSM [LEC], Trauma History Questionnaire [THQ]) AND the same method of assessment was utilised in cases and controls
- ◯ High risk of bias - Trauma exposure not determined according to DSM/ICD criteria OR method to determine trauma exposure not well described or validated OR different method of assessment utilised in cases and controls
- ◯ Unclear risk of bias - Insufficient information to inform judgement (e.g. trauma exposure not ascertained or no clear information regarding methodology)

Rationale for rating given (can include quotes from the text and reviewer explanations for rating given) ____

2. PTSD case ascertainment
Was PTSD case status based on DSM/ICD criteria and assessed using a valid and reliable measure (e.g. structured diagnostic interview) and was the same method of ascertainment utilised in cases and controls?

- ◯ Low risk of bias -PTSD diagnostic status was based on DSM/ICD criteria AND assessed with a validated assessment measure, such as a structured diagnostic interview (e.g. Clinician-Administered PTSD Scale for DSM [CAPS], Structured Clinical Interview for DSM disorder [SCID]) OR based on a specialist clinician (psychiatrist or psychologist) diagnosis utilising DSM/ICD criteria OR a self-report measure with proven validity and reliability as compared to the gold-standard evaluation (e.g. PTSD Checklist for DSM [PCL], Davidson Trauma Scale [DTS], AND the same method of assessment was utilised in cases and controls
- ◯ High risk of bias - PTSD diagnostic status was not determined according to DSM/ICD criteria OR method to determine trauma exposure not well described or validated (e.g. measure not validated or self-reported presence or absence of PTSD) OR different method of assessment utilised in cases and controls
- ◯ Unclear risk of bias - Insufficient information to inform judgement (e.g. no clear information regarding methodology)

Rationale for rating given (can include quotes from the text and reviewer explanations for rating given)

____

3. Inclusion and exclusion criteria
Were inclusion and exclusion factors applied appropriately and uniformly to cases and controls? [certain inclusion/exclusion factors may be specific to diagnostic group e.g. a lifetime history of PTSD as an exclusion factor in controls, but not cases]

- ◯ Low risk of bias - Critical inclusion/exclusion criteria were stated and were applied uniformly to cases and controls, as appropriate (e.g. use of steroid-containing medications an exclusion factor in both cases and controls)
- ◯ High risk of bias - Inclusion/exclusion criteria were vague or unclear OR were not applied uniformly to cases and controls, as appropriate (e.g. use of steroid-containing medications an exclusion factor in controls, but not in cases)
- ◯ Unclear risk of bias - Insufficient information to inform judgement (e.g. inclusion and exclusion criteria not stated)

Rationale for rating given (can include quotes from the text and reviewer explanations for rating given)

____

4. Representative cases and controls
Were cases and controls recruited in an equivalent manner and adequately represent the population being studied?

- ◯ Low risk of bias - Consecutive or random sample of cases clearly representative of PTSD patients (e.g. all PTSD patients in a catchment area) and controls from a similar community setting as patients (e.g. both cases and controls sourced from military veterans) and recruitment was done in an equivalent manner or differing recruitment strategies unlikely to influence outcomes (e.g. community controls sourced through alternative routes, but well matched to patients)
- ◯ High risk of bias - Sample of cases not obtained in a consecutive or random method and not clearly representative of PTSD patients (e.g. patients selected with rare features, such as severe dissociative or psychotic symptoms) OR controls not from a similar community setting as patients (e.g. other hospital patients or living in a different region) OR differing recruitment strategies that are likely to influence results were used (e.g. medical staff used as controls for community patients)
- ◯ Unclear risk of bias - Insufficient information to inform judgement (e.g. recruitment and sampling strategies not clearly stated)

Rationale for rating given (can include quotes from the text and reviewer explanations for rating given)

____

Performance bias

Performance bias refers to systematic differences between groups in the care that is provided, or in exposure to factors other than the interventions of interest

5. Confounding<br>Were confounding factors assessed for using standard, valid and reliable measures used consistently across all study participants and were confounding factors appropriately dealt with	◯ Low risk of bias - Major potential confounding factors (e.g. age, gender, comorbidity, medication use) were assessed for utilising validated measures used consistently in all participants (e.g. diagnostic interview for psychiatric comorbidity in both cases and controls) AND appropriately controlled for (e.g. similar between cases and controls, or controlled for in analysis [e.g. multivariate or subgroup analysis])<br>◯ High risk of bias - Major potential confounding factors (e.g. age, gender, comorbidity, medication use) were not assessed for OR were not assessed using validated measures consistently in all participants (e.g. self-proclaimed 'healthy' status in controls versus diagnostic interview in cases) OR were not appropriately controlled for (e.g. confounders handled differently in cases or controls or not accounted for in analysis)<br>◯ Unclear risk of bias - Insufficient information to inform judgement
Rationale for rating given (can include quotes from the text and reviewer explanations for rating given)	__________________________________

Attrition bias

Attrition bias refers to systematic differences between groups in withdrawals from a study

6. Incomplete outcome data<br>Were there few concerns regarding attrition and was missing data handled appropriately?	◯ Low risk of bias (any missing data unlikely to influence study outcomes) - No clear factors influencing attrition (e.g. response rates adequate and similar for cases and controls, no difference in dropout or exclusion between cases and controls) AND no or very limited (e.g. < 10%) missing outcome data and appropriate method utilized to handle missing outcome data (e.g. appropriate imputation method used)<br>◯ High risk of bias (missing data likely to influence study outcomes) - Clear factors influencing attrition (e.g. response rate unsatisfactory or different between cases and controls, large number lost to follow-up or different between cases and controls) OR a large proportion of missing data (e.g. > 15%) and missing data not handled appropriately (e.g. no or inappropriate imputation method used)<br>◯ Unclear risk of bias - Insufficient information (regarding attrition or missing data) to inform judgement
Rationale for rating given (can include quotes from the text and reviewer explanations for rating given)	__________________________________

Detection bias

Detection bias refers to systematic differences between groups in how outcomes are determined

7. Time period between exposure and outcome<br>Was the time period between trauma exposure and outcome assessment (PTSD case ascertainment and cortisol levels obtained) at least a month and similar in PTSD cases and controls?<br>[does not apply to trauma unexposed controls]	◯ Low risk of bias - Time period between trauma exposure and assessment was at least a month and equivalent between groups (PTSD cases and controls) OR if time period since trauma exposure was not equivalent this was adequately controlled for in analysis<br>◯ High risk of bias - Time period between trauma exposure and assessment was less than a month OR was not equivalent between groups (PTSD cases and controls)<br>◯ Unclear risk of bias - Insufficient information to inform judgement (e.g. time period since trauma exposure not specified)
Rationale for rating given (can include quotes from the text and reviewer explanations for rating given)	__________________________________
8. Outcome assessments<br>Were outcomes (cortisol levels) assessed in a standard, valid and reliable method across all study participants and were assessors blinded to the case status (patient or control)?<br>[As cortisol is measured utilising objective quantitative assessment methods blinding is unlikely to influence outcome (cortisol levels) in most instances]	◯ Low risk of bias - Methods to obtain samples and determine cortisol levels adequately described, validated and performed consistently in cases and controls and blinding ensured or blinding status unlikely to influence outcomes<br>◯ High risk of bias - Methods to obtain samples or determine cortisol levels unclear or vague and performed differently in cases and controls or lack of blinding and absence of blinding likely to influence outcome status<br>◯ Unclear risk of bias - Insufficient information to inform judgement (e.g. cortisol ascertainment methods not described in the study)
Rationale for rating given (can include quotes from the text and reviewer explanations for rating given)	__________________________________
9. Statistical analysis<br>Were appropriate statistical analysis methods used?	◯ Low risk of bias - the statistical analysis approach was clearly described and was appropriate for the study outcomes (e.g. multivariate analysis performed, corrected for multiple testing)<br>◯ High risk of bias - the statistical analysis was not adequately described or was not clearly appropriate (e.g. only univariate statistics performed)<br>◯ Unclear risk of bias - Insufficient information to inform judgement (e.g. statistical analysis approach only partially described)
Rationale for rating given (can include quotes from the text and reviewer explanations for rating given)	__________________________________

Reporting bias

Reporting bias refers to systematic differences between reported and unreported findings

10. Selective reporting<br>Were the outcomes examined prespecified and are all the outcomes reported on (i.e. no evidence of selective outcome reporting)?	◯ Low risk of bias - All the outcomes were clearly prespecified (e.g. in the methods) and all stated outcomes are reported on<br>◯ High risk of bias - Outcomes were not prespecified or not all prespecified outcomes were reported on or additional outcomes that were not prespecified were reported on<br>◯ Unclear risk of bias - Insufficient information to inform judgement
Rationale for rating given (can include quotes from the text and reviewer explanations for rating given)	__________________________________

Other biases

11. Conflict of interest and funding<br>Were there funding sources or other potential sources of conflict of interest that may have influenced the study outcomes?	◯ Low risk of bias - Funding sources and the role played by funders and presence or absence of conflicts of interest explicitly stated and unlikely to influence study outcomes (e.g. funders unlikely to have a vested interest in a specific outcome or conflict of interest not related to the specific study)<br>◯ High risk of bias - Funding sources and the role played by funders and presence of absence of conflicts of interest stated and potential to influence study outcomes exists (e.g. funders likely to have a vested interest in a specific outcome or motivations may exist for investigator(s) to desire outcomes supporting their ideas or beliefs)<br>◯ Unclear risk of bias - Insufficient information to inform judgement (e.g. no explicit statement regarding funding sources or conflicts of interest)
Rationale for rating given (can include quotes from the text and reviewer explanations for rating given)	__________________________________
Any other potential soruces of bias or factors that may influence risk of bias assessment	__________________________________
Notes	__________________________________

Participants

1. Total number of participants

____

Notes for above (can include quotes from the text, location in the text and reviewer explanations)

____

2. Participation agreement or loss to follow-up, mortality, withdrawals and exclusions

____

Notes for above (can include quotes from the text, location in the text and reviewer explanations)

____

PTSD patients
Description as stated in report/paper. Can include quotes from the text.
If data for total sample described - add total sample data and clearly distinguish total sample data from PTSD patient data

3. Description of PTSD patients

____

Notes for above (can include quotes from the text, location in the text and reviewer explanations)

____

4. Number of PTSD patients
(or total PTSD pop. at start of study)

____

Notes for above (can include quotes from the text, location in the text and reviewer explanations)

____

5. Age of PTSD patients

____

Notes for above (can include quotes from the text, location in the text and reviewer explanations)

____

6. Sex of PTSD patients

____

Notes for above (can include quotes from the text, location in the text and reviewer explanations)

____

7. Race/ethnicity of PTSD patients

____

Notes for above (can include quotes from the text, location in the text and reviewer explanations)	__________________________________
8. Index trauma in PTSD patients (any descriptions included in report)	____________________________
Notes for above (can include quotes from the text, location in the text and reviewer explanations)	__________________________________
9. Trauma severity in PTSD patients (e.g. number of lifetime traumas, number of types of traumas)	____________________________
Notes for above (can include quotes from the text, location in the text and reviewer explanations)	__________________________________
10 Time since index trauma in PTSD patients	____________________________
Notes for above (can include quotes from the text, location in the text and reviewer explanations)	__________________________________
11. Developmental stage during which trauma exposure occured in patients (e.g. childhood, adolescence, adult)	____________________________
Notes for above (can include quotes from the text, location in the text and reviewer explanations)	__________________________________
12. PTSD severity in patients	____________________________
Notes for above (can include quotes from the text, location in the text and reviewer explanations)	__________________________________
13. Duration of PTSD /symptoms in patients	____________________________
Notes for above (can include quotes from the text, location in the text and reviewer explanations)	__________________________________
14. Prior or current treatment received for PTSD	____________________________
Notes for above (can include quotes from the text, location in the text and reviewer explanations)	__________________________________
15. Any medical/somatic comorbidities in PTSD patients	____________________________

Notes for above (can include quotes from the text, location in the text and reviewer explanations)	__________
16. Any psychiatric/mental health comorbidities in PTSD patients	__________
Notes for above (can include quotes from the text, location in the text and reviewer explanations)	__________
17. Any substance use or substance use disorders in PTSD patients	__________
Notes for above (can include quotes from the text, location in the text and reviewer explanations)	__________
18. Any current treatment/medication use in PTSD patients (in particular note steroid medications e.g. prednisone and psychiatric medications)	__________
Notes for above (can include quotes from the text, location in the text and reviewer explanations)	__________
19. Any physical parameters noted in patients e.g. BMI, blood pressure)	__________
Notes for above (can include quotes from the text, location in the text and reviewer explanations)	__________
20. Other relevant sociodemographics in PTSD patients	__________
Notes for above (can include quotes from the text, location in the text and reviewer explanations)	__________
21. Any PTSD subgroups measured/reported	__________
Notes for above (can include quotes from the text, location in the text and reviewer explanations)	__________

PTSD controls
Description as stated in report/paper. Can include quotes from the text.
Add data for all control groups included and clearly distinguish between different controls groups

22. Description of controls ______________________________

Notes for above (can include quotes from the text, location in the text and reviewer explanations) ______________________________

23. Number of control participants (or total pop. at start of study) __________________________

Notes for above (can include quotes from the text, location in the text and reviewer explanations) ______________________________

24. Age of control participants __________________________

Notes for above (can include quotes from the text, location in the text and reviewer explanations) ______________________________

25. Sex of control participants __________________________

Notes for above (can include quotes from the text, location in the text and reviewer explanations) ______________________________

26. Race/ethnicity of control participants __________________________

Notes for above (can include quotes from the text, location in the text and reviewer explanations) ______________________________

27. Index trauma in control participants (any descriptions included in report) __________________________

Notes for above (can include quotes from the text, location in the text and reviewer explanations) ______________________________

28. Trauma severity in control participants (e.g. number of lifetime traumas, number of types of traumas) __________________________

Notes for above (can include quotes from the text, location in the text and reviewer explanations) ______________________________

29. Time since index trauma in controls

Notes for above (can include quotes from the text, location in the text and reviewer explanations)

30. Developmental stage during which trauma exposure occured in controls (e.g. childhood, adolescence, adult)

Notes for above (can include quotes from the text, location in the text and reviewer explanations)

31. PTSD symptom severity in controls (often symptom severity will be reported in trauma exposed controls)

Notes for above (can include quotes from the text, location in the text and reviewer explanations)

32. Duration of PTSD symptoms in controls (sometimes stated for trauma exposed controls)

Notes for above (can include quotes from the text, location in the text and reviewer explanations)

33. Any medical/somatic comorbidities in controls

Notes for above (can include quotes from the text, location in the text and reviewer explanations)

34. Any psychiatric/mental health comorbidities in controls

Notes for above (can include quotes from the text, location in the text and reviewer explanations)

35. Any substance use or substance use disorders in controls

Notes for above (can include quotes from the text, location in the text and reviewer explanations)

36. Any current treatment/medication use in controls (in particular note steroid medications e.g. prednisone)

Notes for above (can include quotes from the text, location in the text and reviewer explanations) __________________________________

37. Other relevant sociodemographics in controls ___________________________

Notes for above (can include quotes from the text, location in the text and reviewer explanations) __________________________________

38. Any physical parameters noted in controls e.g. BMI, blood pressure) ___________________________

Notes for above (can include quotes from the text, location in the text and reviewer explanations) __________________________________

Notes: __________________________________

Outcomes

Cortisol measurement
Description as stated in report/paper. Can include quotes from the text.

1. Tissue types cortisol was measured in (tick all that apply)
- ☐ Plasma
- ☐ Serum
- ☐ Whole blood
- ☐ Saliva
- ☐ Urine
- ☐ Hair
- ☐ Nails
- ☐ Other
- ☐ Not specified/unclear

Notes for above (can include quotes from the text, location in the text and reviewer explanations) ______________________

2. Cortisol measured at single or multiple time points (e.g. multiple measurements in a single day or measurements repeated after a few weeks)
- ◯ Single time point
- ◯ Multiple time points
- ◯ Unclear/not stated

Notes for above (can include quotes from the text, location in the text and reviewer explanations) ______________________

3. Time period reflected by sample(s) (e.g. time of day; 24 hours; 1 month) ______________________

Notes for above (can include quotes from the text, location in the text and reviewer explanations) ______________________

4. Method(s) used to obtain samples from participants ______________________

Notes for above (can include quotes from the text, location in the text and reviewer explanations) ______________________

5. Sampling method standardised?
- ◯ Yes
- ◯ No
- ◯ Uncertain/unclear

Notes for above (can include quotes from the text, location in the text and reviewer explanations) ______________________

6. Method(s) used to analyse samples (in laboratory) ______________________

Notes for above (can include quotes from the text, location in the text and reviewer explanations)	____________________________________
7. Analysis method standardised and validated?	◯ Yes<br>◯ No<br>◯ Uncertain/unclear
Notes for above (can include quotes from the text, location in the text and reviewer explanations)	____________________________________
8. Missing cortisol level data and imputation of missing data	____________________________________
Notes for above (can include quotes from the text, location in the text and reviewer explanations)	____________________________________
Notes	____________________________________

Results

Results<br>Include comparative information for each group (i.e. controls) if available.<br>Description as stated in report/paper. Can include quotes from the text.	
1. Comparison made in cortisol levels for which groups	______________________
Notes for above (can include quotes from the text, location in the text and reviewer explanations)	______________________
2. Any subgroups	__________________
Notes for above (can include quotes from the text, location in the text and reviewer explanations)	______________________
3. Number of time points cortisol levels reported for (e.g. am and pm measures obtained or 6 weeks and 10 weeks post trauma)	______________________
Notes for above (can include quotes from the text, location in the text and reviewer explanations)	______________________
4. Length of timepoints reported (e.g. measure representing 24hrs or 2 weeks)	______________________
Notes for above (can include quotes from the text, location in the text and reviewer explanations)	______________________
5. Unit of measurement (e.g. mmol/l or mg/dl)	__________________
Notes for above (can include quotes from the text, location in the text and reviewer explanations)	______________________
6. Reference scales for cortisol (e.g. upper and lower limits)	__________________
Notes for above (can include quotes from the text, location in the text and reviewer explanations)	______________________

Cortisol level in PTSD patients
Include comparative information for each group (i.e. subgroups) if available.
Description as stated in report/paper. Can include quotes from the text.

7. Mean (M) cortisol level patients ____________________

Notes for above (can include quotes from the text, location in the text and reviewer explanations) ____________________

8. Standard deviation (SD) PTSD patients ____________________

Notes for above (can include quotes from the text, location in the text and reviewer explanations) ____________________

9. Number (N) of PTSD patients cortisol measured in ____________________

Notes for above (can include quotes from the text, location in the text and reviewer explanations) ____________________

10. Other statistics reported regarding cortisol levels in PTSD patients (E.g. medians (mdn), standard errors (SE), confidence intervals (CI), interquartile ranges (IQR) and ranges) ____________________

Notes for above (can include quotes from the text, location in the text and reviewer explanations) ____________________

Cortisol level in controls
Include comparative information for each group (i.e. controls) if available.
Description as stated in report/paper. Can include quotes from the text.

11. Mean (M) cortisol level controls ____________________

Notes for above (can include quotes from the text, location in the text and reviewer explanations) ____________________

12. Standard deviation (SD) controls ____________________

Notes for above (can include quotes from the text, location in the text and reviewer explanations) ____________________

13. Number (N) of controls cortisol measured in ____________________

Notes for above (can include quotes from the text, location in the text and reviewer explanations) __________________________________

14. Other statistics reported regarding cortisol levels in controls (E.g. medians (mdn), standard errors (SE), confidence intervals (CI), interquartile ranges (IQR) and ranges) __________________________________

Notes for above (can include quotes from the text, location in the text and reviewer explanations) __________________________________

Statistical analysis

15. No. participants (patients and controls) for which cortisol levels are missing for and reasons __________________________________

Notes for above (can include quotes from the text, location in the text and reviewer explanations) __________________________________

16. No. participants moved from other group and reasons (e.g. in cohort studies patient and control status may change at different time points) __________________________________

Notes for above (can include quotes from the text, location in the text and reviewer explanations) __________________________________

17. Any other results reported __________________________________

Notes for above (can include quotes from the text, location in the text and reviewer explanations) __________________________________

18. Statistical methods used and appropriateness of these methods
(e.g. multivariate analysis) __________________________________

Notes for above (can include quotes from the text, location in the text and reviewer explanations) __________________________________

19. Confounding factors/ effect modifiers accounted for in the analyses __________________________________

Notes for above (can include quotes from the text, location in the text and reviewer explanations) __________________________________

20. Reanalysis required?<br>(specify why)	◯ Yes<br>◯ No<br>◯ Unclear/unsure
Notes for above (can include quotes from the text, location in the text and reviewer explanations)	__________________________
21. Reanalysis possible?<br>(specify why)	◯ Yes<br>◯ No<br>◯ Unclear/unsure
Notes for above (can include quotes from the text, location in the text and reviewer explanations)	__________________________
Notes	__________________________

Applicability

1. Have important populations been excluded from the study?	◯ Yes<br>◯ No<br>◯ Unclear/unsure
Notes (can include quotes from the text, location in the text and reviewer explanations)	__________________________________
2. Does the study directly address the review question?<br>(any issues of partial or indirect applicability)	◯ Yes<br>◯ No<br>◯ Unclear/unsure
Notes (can include quotes from the text, location in the text and reviewer explanations)	__________________________________
Notes	__________________________________

Other Information

**Other information**<br>**Descriptions as stated in report/paper. Can include quotes from the text.**	
1. Study limitations	__________________
Notes for above (can include quotes from the text, location in the text and reviewer explanations)	__________________
2. Key conclusions of the study authors	__________________
Notes for above (can include quotes from the text, location in the text and reviewer explanations)	__________________
3. References to other relevant studies	__________________
4. Correspondence required for further study information<br>(what and from whom)	__________________
5. Further study information requested<br>(from whom, what and when)	__________________
6. Correspondence received<br>(from whom, what and when)	__________________
Notes	__________________
