# Supplementary material for: Cortisol levels in different tissue samples in posttraumatic stress disorder patients versus controls: a systematic review and meta-analysis protocol
Source: Syst Rev. 2019 Jan 7;8:7. doi: 10.1186/s13643-018-0936-x (PMC6322257; doi:10.1186/s13643-018-0936-x)
Supplement: Supplementary file 6 — Modified Newcastle Ottawa Scale. (PDF 40 kb) [file 13643_2018_936_MOESM6_ESM.pdf]

# Modified Newcastle-Ottawa Scale (NOS)

Record ID __________________________________

## Domain of evaluation: Methods for selecting study participants (i.e. Selection bias)

Is the source population (cases, controls, cohorts) appropriate and representative of the population of interest?
Example of low risk of bias: A consecutive sample or random selection from a population that is representative of the condition under study.
Example of moderate risk of bias: A consecutive sample or random selection from a population that is not highly representative of the condition under study.
Example of high risk of bias: The source population cannot be defined or enumerated (i.e. volunteering or self-recruitment).

- ◯ Definitely no (high risk of bias)
- ◯ Mostly no
- ◯ Mostly yes
- ◯ Definitely yes (low risk of bias)

## Domain of evaluation: Methods to control confounding (i.e. Performance bias)

Is the sample size adequate and is there sufficient power to detect a meaningful difference in the outcome of interest?
Example of low risk of bias: Sample size was adequate and there was sufficient power to detect a difference in the outcome.
Example of high risk of bias: Sample size was small and there was not enough power to test outcome of interest.

- ◯ Definitely no (high risk of bias)
- ◯ Mostly no
- ◯ Mostly yes
- ◯ Definitely yes (low risk of bias)

---

Did the study identify and adjust for any variables or confounders that may influence the outcome?
Example of low risk of bias: The study identified and adjusted for all possible confounders that may influence estimates of association between exposure and outcome (i.e. Was the patient being treated for a medical condition such as chronic pain and was being prescribed opioids while on methadone treatment?)
Example of moderate risk of bias: The study identified and reported possible variables that may influence the outcome but did not explore the interaction.
Example of high risk of bias: The study either did not report any variables of influence or acknowledge variables of influence when it was clear they were present.

- ◯ Definitely no (high risk of bias)
- ◯ Mostly no
- ◯ Mostly yes
- ◯ Definitely yes (low risk of bias)

## Domain of evaluation: Statistical methods (i.e. Detection bias)

Did the study use appropriate statistical analysis methods relative to the outcome of interest?
Example of low risk of bias: The study reported use of appropriate statistical analysis as required (i.e. adjusting for an unbalanced distribution of a specific covariate among sexes, or correcting for multiple testing error)
Example of moderate risk of bias: The study either used correct statistical methods but did not report them well, or used the incorrect methods but reported them in detail.
Example of high risk of bias: The study did not use appropriate statistical analysis as required (i.e. did not adjust for an unbalanced distribution of a specific covariate among sexes, or correct for multiple testing error when necessary) or did not report them adequately.

- ◯ Definitely no (high risk of bias)
- ◯ Mostly no
- ◯ Mostly yes
- ◯ Definitely yes (low risk of bias)

Is there little missing data and did the study handle it accordingly?
Example of low risk of bias: The study acknowledged missing data to be less than 10% and specified the method of handling it.
Example of moderate risk of bias: The study either had greater than 15% but they specified the method they used to handle it.
Example of high risk of bias: The study had greater than 15% missing data and did not handle it at all.

- ◯ Definitely no (high risk of bias)
- ◯ Mostly no
- ◯ Mostly yes
- ◯ Definitely yes (low risk of bias)

## Domain of evaluation: Methods for measuring outcome variables (i.e. Information bias)

Is the methodology of the outcome measurement explicitly stated and is it appropriate?
Example of low risk of bias: The study provides a detailed description of the outcome measure(s) which are appropriate for the outcome of interest.
Example of moderate risk of bias: The study provides a somewhat complete description of outcome measurements and they are justified.
Example of high risk of bias: The study provides limited information on the methods of measuring the outcome and the measure is not appropriate considering the outcome.

- ◯ Definitely no (high risk of bias)
- ◯ Mostly no
- ◯ Mostly yes
- ◯ Definitely yes (low risk of bias)

Is there an objective assessment of the outcome of interest?
Example of low risk of bias: The study used objective methods to discern the outcome status of participants (i.e. laboratory measurements, medical records).
Example of moderate risk of bias: The study relied on subjective data as the primary method to discern outcome status of participants (i.e. self-report).
Example of high risk of bias: The study had limited reporting about assessment of outcomes.

- ◯ Definitely no (high risk of bias)
- ◯ Mostly no
- ◯ Mostly yes
- ◯ Definitely yes (low risk of bias)
