# Supplementary material for: Cortisol levels in different tissue samples in posttraumatic stress disorder patients versus controls: a systematic review and meta-analysis protocol
Source: Syst Rev. 2019 Jan 7;8:7. doi: 10.1186/s13643-018-0936-x (PMC6322257; doi:10.1186/s13643-018-0936-x)
Supplement: Supplementary file 7 — Risk of bias (ROB) assessment. (PDF 50 kb) [file 13643_2018_936_MOESM7_ESM.pdf]

Risk of bias (ROB) assessment

Record ID

____

Reviewer initials:

____

Date form completed

____
(dd/mm/yyyy)

Selection bias
Selection bias refers to systematic differences between baseline characteristics of the groups that are compared

1. Ascertainment of trauma exposure
Was trauma exposure based on DSM/ICD criteria and assessed using a valid and reliable measure and was the same method of ascertainment utilised in cases and controls?

- ◯ Low risk of bias - Trauma exposure was based on DSM/ICD criteria AND assessed with a validated trauma assessment measure (e.g. Life Events Checklist for DSM [LEC], Trauma History Questionnaire [THQ]) AND the same method of assessment was utilised in cases and controls
- ◯ High risk of bias - Trauma exposure not determined according to DSM/ICD criteria OR method to determine trauma exposure not well described or validated OR different method of assessment utilised in cases and controls
- ◯ Unclear risk of bias - Insufficient information to inform judgement (e.g. trauma exposure not ascertained or no clear information regarding methodology)

Rationale for rating given (can include quotes from the text and reviewer explanations for rating given)

____

2. PTSD case ascertainment
Was PTSD case status based on DSM/ICD criteria and assessed using a valid and reliable measure (e.g. structured diagnostic interview) and was the same method of ascertainment utilised in cases and controls?

- ◯ Low risk of bias -PTSD diagnostic status was based on DSM/ICD criteria AND assessed with a validated assessment measure, such as a structured diagnostic interview (e.g. Clinician-Administered PTSD Scale for DSM [CAPS], Structured Clinical Interview for DSM disorder [SCID]) OR based on a specialist clinician (psychiatrist or psychologist) diagnosis utilising DSM/ICD criteria OR a self-report measure with proven validity and reliability as compared to the gold-standard evaluation (e.g. PTSD Checklist for DSM [PCL], Davidson Trauma Scale [DTS], AND the same method of assessment was utilised in cases and controls
- ◯ High risk of bias - PTSD diagnostic status was not determined according to DSM/ICD criteria OR method to determine trauma exposure not well described or validated (e.g. measure not validated or self-reported presence or absence of PTSD) OR different method of assessment utilised in cases and controls
- ◯ Unclear risk of bias - Insufficient information to inform judgement (e.g. no clear information regarding methodology)

Rationale for rating given (can include quotes from the text and reviewer explanations for rating given) __________________________________

3. Inclusion and exclusion criteria
Were inclusion and exclusion factors applied appropriately and uniformly to cases and controls? [certain inclusion/exclusion factors may be specific to diagnostic group e.g. a lifetime history of PTSD as an exclusion factor in controls, but not cases]

- ◯ Low risk of bias - Critical inclusion/exclusion criteria were stated and were applied uniformly to cases and controls, as appropriate (e.g. use of steroid-containing medications an exclusion factor in both cases and controls)
- ◯ High risk of bias - Inclusion/exclusion criteria were vague or unclear OR were not applied uniformly to cases and controls, as appropriate (e.g. use of steroid-containing medications an exclusion factor in controls, but not in cases)
- ◯ Unclear risk of bias - Insufficient information to inform judgement (e.g. inclusion and exclusion criteria not stated)

Rationale for rating given (can include quotes from the text and reviewer explanations for rating given) __________________________________

4. Representative cases and controls
Were cases and controls recruited in an equivalent manner and adequately represent the population being studied?

- ◯ Low risk of bias - Consecutive or random sample of cases clearly representative of PTSD patients (e.g. all PTSD patients in a catchment area) and controls from a similar community setting as patients (e.g. both cases and controls sourced from military veterans) and recruitment was done in an equivalent manner or differing recruitment strategies unlikely to influence outcomes (e.g. community controls sourced through alternative routes, but well matched to patients)
- ◯ High risk of bias - Sample of cases not obtained in a consecutive or random method and not clearly representative of PTSD patients (e.g. patients selected with rare features, such as severe dissociative or psychotic symptoms) OR controls not from a similar community setting as patients (e.g. other hospital patients or living in a different region) OR differing recruitment strategies that are likely to influence results were used (e.g. medical staff used as controls for community patients)
- ◯ Unclear risk of bias - Insufficient information to inform judgement (e.g. recruitment and sampling strategies not clearly stated)

Rationale for rating given (can include quotes from the text and reviewer explanations for rating given)

____

Performance bias

Performance bias refers to systematic differences between groups in the care that is provided, or in exposure to factors other than the interventions of interest

5. Confounding
Were confounding factors assessed for using standard, valid and reliable measures used consistently across all study participants and were confounding factors appropriately dealt with

- ◯ Low risk of bias - Major potential confounding factors (e.g. age, gender, comorbidity, medication use) were assessed for utilising validated measures used consistently in all participants (e.g. diagnostic interview for psychiatric comorbidity in both cases and controls) AND appropriately controlled for (e.g. similar between cases and controls, or controlled for in analysis [e.g. multivariate or subgroup analysis])
- ◯ High risk of bias - Major potential confounding factors (e.g. age, gender, comorbidity, medication use) were not assessed for OR were not assessed using validated measures consistently in all participants (e.g. self-proclaimed 'healthy' status in controls versus diagnostic interview in cases) OR were not appropriately controlled for (e.g. confounders handled differently in cases or controls or not accounted for in analysis)
- ◯ Unclear risk of bias - Insufficient information to inform judgement

Rationale for rating given (can include quotes from the text and reviewer explanations for rating given)

____

Attrition bias

Attrition bias refers to systematic differences between groups in withdrawals from a study

6. Incomplete outcome data
Were there few concerns regarding attrition and was missing data handled appropriately?

◯ Low risk of bias (any missing data unlikely to influence study outcomes) - No clear factors influencing attrition (e.g. response rates adequate and similar for cases and controls, no difference in dropout or exclusion between cases and controls) AND no or very limited (e.g. < 10%) missing outcome data and appropriate method utilized to handle missing outcome data (e.g. appropriate imputation method used)
◯ High risk of bias (missing data likely to influence study outcomes) - Clear factors influencing attrition (e.g. response rate unsatisfactory or different between cases and controls, large number lost to follow-up or different between cases and controls) OR a large proportion of missing data (e.g. > 15%) and missing data not handled appropriately (e.g. no or inappropriate imputation method used)
◯ Unclear risk of bias - Insufficient information (regarding attrition or missing data) to inform judgement

Rationale for rating given (can include quotes from the text and reviewer explanations for rating given)

____

Detection bias

Detection bias refers to systematic differences between groups in how outcomes are determined

7. Time period between exposure and outcome
Was the time period between trauma exposure and outcome assessment (PTSD case ascertainment and cortisol levels obtained) at least a month and similar in PTSD cases and controls?
[does not apply to trauma unexposed controls]

◯ Low risk of bias - Time period between trauma exposure and assessment was at least a month and equivalent between groups (PTSD cases and controls) OR if time period since trauma exposure was not equivalent this was adequately controlled for in analysis
◯ High risk of bias - Time period between trauma exposure and assessment was less than a month OR was not equivalent between groups (PTSD cases and controls)
◯ Unclear risk of bias - Insufficient information to inform judgement (e.g. time period since trauma exposure not specified)

Rationale for rating given (can include quotes from the text and reviewer explanations for rating given)

____

8. Outcome assessments
Were outcomes (cortisol levels) assessed in a standard, valid and reliable method across all study participants and were assessors blinded to the case status (patient or control)?
[As cortisol is measured utilising objective quantitative assessment methods blinding is unlikely to influence outcome (cortisol levels) in most instances]

- ◯ Low risk of bias - Methods to obtain samples and determine cortisol levels adequately described, validated and performed consistently in cases and controls and blinding ensured or blinding status unlikely to influence outcomes
- ◯ High risk of bias - Methods to obtain samples or determine cortisol levels unclear or vague and performed differently in cases and controls or lack of blinding and absence of blinding likely to influence outcome status
- ◯ Unclear risk of bias - Insufficient information to inform judgement (e.g. cortisol ascertainment methods not described in the study)

Rationale for rating given (can include quotes from the text and reviewer explanations for rating given)

____

9. Statistical analysis
Were appropriate statistical analysis methods used?

- ◯ Low risk of bias - the statistical analysis approach was clearly described and was appropriate for the study outcomes (e.g. multivariate analysis performed, corrected for multiple testing)
- ◯ High risk of bias - the statistical analysis was not adequately described or was not clearly appropriate (e.g. only univariate statistics performed)
- ◯ Unclear risk of bias - Insufficient information to inform judgement (e.g. statistical analysis approach only partially described)

Rationale for rating given (can include quotes from the text and reviewer explanations for rating given)

____

Reporting bias

Reporting bias refers to systematic differences between reported and unreported findings

10. Selective reporting
Were the outcomes examined prespecified and are all the outcomes reported on (i.e. no evidence of selective outcome reporting)?

- ◯ Low risk of bias - All the outcomes were clearly prespecified (e.g. in the methods) and all stated outcomes are reported on
- ◯ High risk of bias - Outcomes were not prespecified or not all prespecified outcomes were reported on or additional outcomes that were not prespecified were reported on
- ◯ Unclear risk of bias - Insufficient information to inform judgement

Rationale for rating given (can include quotes from the text and reviewer explanations for rating given)

____

Other biases

11. Conflict of interest and funding<br>Were there funding sources or other potential sources of conflict of interest that may have influenced the study outcomes?	◯ Low risk of bias - Funding sources and the role played by funders and presence or absence of conflicts of interest explicitly stated and unlikely to influence study outcomes (e.g. funders unlikely to have a vested interest in a specific outcome or conflict of interest not related to the specific study)<br>◯ High risk of bias - Funding sources and the role played by funders and presence of absence of conflicts of interest stated and potential to influence study outcomes exists (e.g. funders likely to have a vested interest in a specific outcome or motivations may exist for investigator(s) to desire outcomes supporting their ideas or beliefs)<br>◯ Unclear risk of bias - Insufficient information to inform judgement (e.g. no explicit statement regarding funding sources or conflicts of interest)
Rationale for rating given (can include quotes from the text and reviewer explanations for rating given)	__________________________________
Any other potential soruces of bias or factors that may influence risk of bias assessment	__________________________________
Notes	__________________________________
