# Supplementary material for: Cortisol levels in different tissue samples in posttraumatic stress disorder patients versus controls: a systematic review and meta-analysis protocol
Source: Syst Rev. 2019 Jan 7;8:7. doi: 10.1186/s13643-018-0936-x (PMC6322257; doi:10.1186/s13643-018-0936-x)
Supplement: Supplementary file 8 — Example risk of bias (ROB) figure. (DOCX 13 kb) [file 13643_2018_936_MOESM8_ESM.docx]

**Additional file 8: Example risk of bias (ROB) figure**

|  | Selection bias | | | | Performance bias | Attrition bias | Detection bias | | | Reporting bias | Other biases |  |
| --- | --- | --- | --- | --- | --- | --- | --- | --- | --- | --- | --- | --- |
| Study details | Ascertainment of trauma exposure | PTSD case ascertainment | Inclusion and exclusion criteria | Representative cases and controls | Confounding | Incomplete outcome data | Time period between exposure and outcome | Outcome assessments | Statistical analysis | Selective reporting | Conflict of interest and funding | Comments |
| Johnson et al., 2014 | + | - | + | ? | - | + | ? | + | + | - | ? | Study only included trauma unexposed controls |
| Smith et al., 2001 | - | + | - | + | + | ? | - | + | - | ? | + |  |
|  |  |  | | | | | | | | | |  |
|  | + | Low risk of bias | | | | | | | | | |  |
|  | - | High risk of bias | | | | | | | | | |  |
|  | ? | Unclear risk of bias | | | | | | | | | |  |
