# Supplementary material for: Cortisol levels in different tissue samples in posttraumatic stress disorder patients versus controls: a systematic review and meta-analysis protocol
Source: Syst Rev. 2019 Jan 7;8:7. doi: 10.1186/s13643-018-0936-x (PMC6322257; doi:10.1186/s13643-018-0936-x)
Supplement: Supplementary file 9 — List of potential moderators. (DOCX 15 kb) [file 13643_2018_936_MOESM9_ESM.docx]

**Additional file 9: List of potential moderators**

| **Study characteristics** | | **To be included in subgroup analysis** | **To be included in meta-regression** | **To be included in sensitivity analysis** | **To be included in qualitative synthesis** |
| --- | --- | --- | --- | --- | --- |
| Study Information | |  |  |  |  |
|  | Study authors |  |  |  | x |
|  | Year of publication |  | x |  | x |
| Population And Setting | |  |  |  |  |
|  | Country |  |  |  | x |
| Study Methods | |  |  |  |  |
|  | Study design |  |  | x | x |
|  | Method used to determine trauma exposure |  |  |  | x |
|  | Method used to determine PTSD diagnostic status |  |  |  | x |
|  | Types of control groups included (TEC and TUC) | x |  |  | x |
| Risk of bias | |  |  |  |  |
|  | Modified NOS total score |  |  | x | x |
|  | Modified NOS domain scores |  |  | x | x |
| Participants | |  |  |  |  |
|  | Number |  |  |  | x |
|  | Age |  | x |  | x |
|  | Sex |  | x |  | x |
|  | Race/ethnicity |  |  |  | x |
|  | Trauma type (e.g. combat, sexual, natural disasters) |  | x |  | x |
|  | Trauma severity |  |  |  | x |
|  | Time since index trauma |  | x |  | x |
|  | Developmental stage of trauma exposure |  | x |  | x |
|  | PTSD severity |  | x |  | x |
|  | PTSD duration |  |  |  | x |
|  | Treatment status |  |  |  | x |
|  | PTSD subgroups (e.g. dissociative subtype) |  |  |  | x |
|  | Somatic comorbidities |  |  |  | x |
|  | Psychiatric comorbidities |  | x |  | x |
|  | Substance use |  |  |  | x |
|  | Medication use |  |  |  | x |
|  | Physical parameters (e.g. BMI, blood pressure) |  |  |  | x |
| Cortisol measurement | |  |  |  |  |
|  | Tissue sample type(s)* |  |  |  | x |
|  | Time or time period of sampling (e.g. time of day for acute measures and length of hair sample representing window for hair sampling) |  | x |  | x |
|  | Method used to determine cortisol levels: ELISA or LC-MS |  | x |  | x |
| * Samples will be organised and analysed according to tissue sample type as this is the main study outcome  BMI, body mass index; ELISA, enzyme-linked immunosorbent assay; LC-MS, Liquid chromatography–mass spectrometry; PTSD, posttraumatic stress disorder; TEC, trauma exposed controls; TUC, trauma unexposed controls | | | | | |
